# Supplementary material for: Splice-Junction-Based Mapping of Alternative Isoforms in the Human Proteome
Source: Cell Rep. Author manuscript; Available in PMC 2020 Jan 15. (PMC6961840; doi:10.1016/j.celrep.2019.11.026)

A

sp|P06753|TPM3_HUMAN|ENSG00000143549|MXE1|2629|chr1|154170469|154170711|−2|r140|T4,sp|P09493|TPM1_
AISEELDHALNDM[15.99]TSG q value: 0.0002918 Tr_novel:TRUE RefSeq_Novel:TRUE
Search result spec prec mz: 859.8814 Actual spec prec mz: 859.88141
Fragments matched per AA: 0.875 Proportion of top 20 peaks matched: 0.45

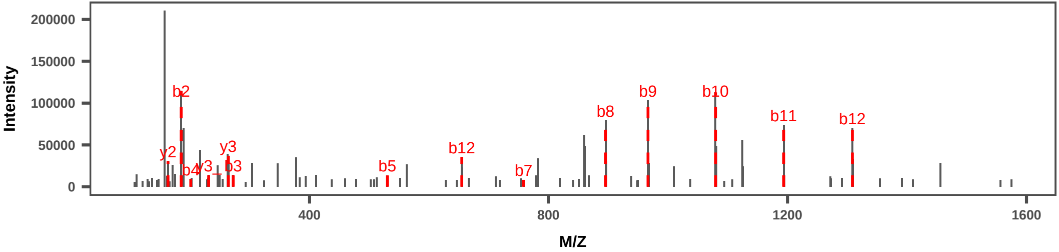

B

Scatterplot of predicted elution time
Fitting R2: 0.889
Novel peptide residual Z score: −0.535
Number of peptides: 969

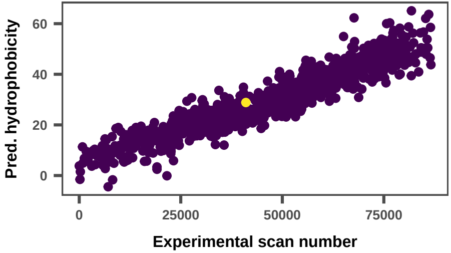

C

Distributions of residuals from best−fit line
of predicted RT vs Expt. scan number
Line: Z score of novel peptide
Z: −0.535

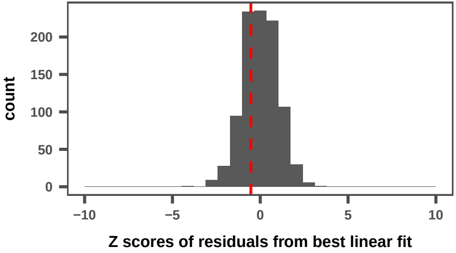

Supplement: 2 [file NIHMS1546469-supplement-2.zip › DF1/PXD006675/VentricularSeptum/VentricularSeptum_22_TPM3_AISEELDHALNDMTSG.pdf]
